# Supplementary material for: The quantum geometric origin of capacitance in insulators
Source: Nat Commun. 2024 May 30;15:4621. doi: 10.1038/s41467-024-48808-x (PMC11139914; doi:10.1038/s41467-024-48808-x)
Supplement: Supplementary file 1 — Supplementary Information [file 41467_2024_48808_MOESM1_ESM.pdf]

Supplementary information for "The quantum geometric origin of capacitance in insulators"

Ilia Komissarov, Tobias Holder, Raquel Queiroz

In this supplementary information, we present a detailed derivation of the relation between interband and intraband conductivity (Sec. I), quantum geometry (Sec. II), and the dynamical polarization (Sec. III). We apply the obtained relations to various example systems: namely, we present in detail how the time-dependent polarization enters in Landau levels (Sec. IV), derive the quantum geometric tensor for Landau levels (Sec. V), and develop some intuition behind these findings by comparing them with a semiclassical point of view (Sec. VI). This is followed by a derivation of the capacitance in a gapped Dirac cone model (Sec. VII). Lastly, we discuss the applications to the dielectric constant and present the complete data which was used to generate Figure 4 of the main text (Sec. VIII).

I. INTERBAND AND INTRABAND CONDUCTIVITY

In this section, we demonstrate how the Kubo formula for conductivity [1] can be split into intraband and interband terms. The former describes the conventional dissipative Fermi-surface transport, whereas the latter arises due to transitions between different bands. The starting point is the standard expression for the conductivity tensor (cf. e. g. [2]):

$$\sigma^{\mu\nu}(\omega) = \frac{i\bar{n}e^2}{m\omega_+}\delta^{\mu\nu} + \frac{1}{\hbar\omega_+ A}\int_0^\infty dt\, e^{i\omega_+ t}\,\langle[\hat{J}^\mu(t), \hat{J}^\nu(0)]\rangle\,, \tag{1}$$

where $\bar{n}$ is the total charge carrier density, m and e are the mass and the charge of the carriers, and A is the area of the conducting sample. The brackets $\langle\ldots\rangle$ denote the vacuum expectation value. The convergence of the integral is ensured by an infinitesimal relaxation rate, i. e. $\omega_+ = \omega + i\varepsilon$, $\varepsilon > 0$. Henceforth, we mostly omit the subscript $+$, restoring it only where necessary to prevent singular behavior.

By inserting the complete basis of energy eigenstates $|m\mathbf{k}\rangle$, we write the commutator in Eq. (1) as

$$\langle[\hat{J}^\mu(t), \hat{J}^\nu(0)]\rangle = \sum_{nm}\int \frac{A\,d^2\mathbf{k}}{(2\pi)^2} f_{nm}(\mathbf{k})\,\langle n\mathbf{k}|\hat{J}^\mu|m\mathbf{k}\rangle\,\langle m\mathbf{k}|\hat{J}^\nu|n\mathbf{k}\rangle\,e^{i\omega_{nm}(\mathbf{k})t}\,, \tag{2}$$

where the indices m and n enumerate the bands, $\omega_{nm} \equiv \omega_n - \omega_m$, and $f_{nm} \equiv f_n - f_m$, where $f_n(\mathbf{k}) = \theta(E_F - \hbar\omega_n(\mathbf{k}))$ is the zero temperature Fermi-Dirac distribution function with respect to the Fermi energy E_F. The matrix elements of the current operators are evaluated in the basis of Bloch states $|n\mathbf{k}\rangle = u_n(\mathbf{k})\,|\mathbf{k}\rangle$, where $u_n(\mathbf{k})$ are the eigenstates of the Hamiltonian in the momentum space: $\hat{H}(\mathbf{k})u_n(\mathbf{k}) = \hbar\omega_n(\mathbf{k})u_n(\mathbf{k})$. In the following, we omit the momentum index and likewise use the shorthand notation of the d-dimensional integral

$$\int_{\mathrm{BZ}} \equiv \int \frac{d^d\mathbf{k}}{(2\pi)^d}\,. \tag{3}$$

Plugging (2) into (1) and evaluating the time-integral, we obtain

$$\sigma^{\mu\nu}(\omega) = -\frac{i}{\hbar}\sum_{nm}\int_{\mathrm{BZ}} \frac{f_{nm}}{\omega_{nm}}\frac{J^\mu_{nm}J^\nu_{mn}}{\omega_{nm}+\omega}\,, \tag{4}$$

where the first (diamagnetic) term in (1) was used to subtract the singular in $\omega \to 0$ limit piece in the commutator term. The remaining sum in Eq. (4) can be split into two contributions: The *intraband* part with $m = n$ and the *interband* part where $m \neq n$. The intraband conductivity is obtained from (4) by substituting

$$\begin{aligned} f_{nm} &\to f\left(E_F - E_n(\mathbf{k}+\mathbf{q})\right) - f\left(E_F - E_n(\mathbf{k})\right)\,, \\ \omega_{nm} &\to \omega_n(\mathbf{k}+\mathbf{q}) - \omega_n(\mathbf{k})\,, \end{aligned} \tag{5}$$

and taking a limit $\mathbf{q} \to 0$

$$\sigma^{\mu\nu}_{\mathrm{intraband}}(\omega) = \frac{i}{\omega}\sum_n \int_{\mathrm{BZ}} f'_n J^\mu_{nn} J^\nu_{nn}\,. \tag{6}$$

At zero temperature, the derivative of the Fermi function is simply a delta function $\delta(E_F - \hbar\omega_n)$ that selects all the points in the BZ where the bands cross the Fermi surface. Hence, this contribution vanishes in insulators in the absence of disorder.

The interband ($m \neq n$) contribution to the sum (4) is convenient to express in terms of the position operators to make an explicit connection with quantum geometric quantities such as Berry curvature and quantum metric. In order to do so, we utilize

$$\begin{aligned} J^{\mu}_{nm} = \frac{e}{\hbar}\langle n|\partial^{\mu}\hat{H}|m\rangle &= \frac{e}{\hbar}\langle n|\,\partial^{\mu}\left(\hat{H}\,|m\rangle\right) - \frac{e}{\hbar}\langle n|\,\hat{H}\,|\partial^{\mu} m\rangle \\ &= \frac{e}{\hbar}(E_m - E_n)\,\langle n|\partial^{\mu} m\rangle = -ie\omega_{nm} r^{\mu}_{nm}\,. \end{aligned} \tag{7}$$

Upon using $J^{\mu}_{nm} = -ie\omega_{nm} r^{\mu}_{nm}$, the interband contribution becomes

$$\sigma^{\mu\nu}_{\text{interband}}(\omega) = -\frac{ie^2}{\hbar}\sum_{n\neq m}\int_{\text{BZ}} f_{nm}\omega_{nm}\frac{r^{\mu}_{nm} r^{\nu}_{mn}}{\omega_{nm} + \omega}\,. \tag{8}$$

The above term does not vanish in insulators even at zero frequency. For example, in the limit $\omega \to 0$ the expression above takes the form of the celebrated TKNN formula [3]

$$\begin{aligned} \sigma^{\mu\nu}_{\text{interband}}(0) &= -\frac{ie^2}{\hbar}\sum_{n\neq m}\int_{\text{BZ}}(f_n - f_m) r^{\mu}_{nm} r^{\nu}_{mn} = -\frac{ie^2}{\hbar}\sum_{n,m}\int_{\text{BZ}} f_n (r^{\mu}_{nm} r^{\nu}_{mn} - r^{\nu}_{nm} r^{\mu}_{mn}) \\ &= -\frac{ie^2}{\hbar}\sum_{n}\int_{\text{BZ}} f_n(\langle n|\hat{r}^{\mu}\hat{r}^{\nu}|n\rangle - \langle n|\hat{r}^{\nu}\hat{r}^{\mu}|n\rangle) = -\frac{ie^2}{\hbar}\sum_{n}\int_{\text{BZ}} f_n(\langle\partial^{\mu} n|\partial^{\nu} n\rangle - \langle\partial^{\nu} n|\partial^{\mu} n\rangle)\,, \end{aligned} \tag{9}$$

i.e. the conductivity is, up to a constant, the sum of the Chern numbers of the occupied bands. At non-zero frequency, on the other hand, it is not obvious that the interband contribution (8) is geometric in its origin: something one would expect to be the case in an insulator, at least in the small ω regime. To investigate this, one may further write:

$$\begin{aligned} \sigma^{\mu\nu}_{\text{interband}}(\omega) &= -\frac{ie^2}{\hbar}\sum_{n\neq m}\int_{\text{BZ}}\left[f_n(1-f_m) - f_m(1-f_n)\right]\,\omega_{nm}\frac{r^{\mu}_{nm} r^{\nu}_{mn}}{\omega_{nm}+\omega} \\ &= -\frac{ie^2}{\hbar}\sum_{n\neq m}\int_{\text{BZ}} f_n(1-f_m)\,\omega_{nm}\left(\frac{r^{\mu}_{nm} r^{\nu}_{mn}}{\omega_{nm}+\omega} - \frac{r^{\nu}_{nm} r^{\mu}_{mn}}{\omega_{nm}-\omega}\right)\,, \end{aligned} \tag{10}$$

where we relabeled the summation indices in the second line. One may proceed by introducing the interband matrix elements of the Berry curvature and the quantum metric

$$\Omega^{\mu\nu}_{nm} = i(r^{\mu}_{nm} r^{\nu}_{mn} - r^{\nu}_{nm} r^{\mu}_{mn})\,, \qquad g^{\mu\nu}_{nm} = \frac{1}{2}(r^{\mu}_{nm} r^{\nu}_{mn} + r^{\nu}_{nm} r^{\mu}_{mn})\,. \tag{11}$$

With the definitions above, interband conductivity reads

$$\sigma^{\mu\nu}_{\text{interband}}(\omega) = \frac{2ie^2}{\hbar}\sum_{n\neq m}\int_{\text{BZ}} f_n(1-f_m)\frac{\omega\omega_{mn}}{\omega^2_{mn} - \omega^2} g^{\mu\nu}_{nm} - \frac{e^2}{\hbar}\sum_{n\neq m}\int_{\text{BZ}} f_n(1-f_m)\frac{\omega^2_{mn}}{\omega^2_{mn} - \omega^2}\Omega^{\mu\nu}_{nm}\,. \tag{12}$$

Note that the second term never contributes to the longitudinal conductivity, since $\Omega^{\mu\nu}_{nm}$ is an antisymmetric tensor. The metric $g^{\mu\nu}_{nm}$, on the other hand, may have off-diagonal components and contribute to $\sigma^{xy}(\omega)$. As we can see, the dispersion-dependent factors in the expression above prevent us from re-summing all the interband transitions into the ground state quantities: Berry curvature and quantum metric

$$\Omega^{\mu\nu} = i\sum_{n\neq m} f_n(1-f_m)(r^{\mu}_{nm} r^{\nu}_{mn} - r^{\nu}_{nm} r^{\mu}_{mn})\,, \qquad g^{\mu\nu} = \frac{1}{2}\sum_{n\neq m} f_n(1-f_m)\,(r^{\mu}_{nm} r^{\nu}_{mn} + r^{\nu}_{nm} r^{\mu}_{mn})\,. \tag{13}$$

This is a consequence of the non-adiabaticity introduced by the presence of ω. Nevertheless, it does not mean that the interband contribution (8) is not purely quantum-geometric. As we will show explicitly in the next section, by considering the geometry of wavefunctions in space-time ($\mathbf{k}$, t), one is able to express (8) solely in terms of the Hilbert space quantities.

II. TIME-DEPENDENT QUANTUM GEOMETRIC TENSOR

In clean insulators, with a finite excitation gap, quasiparticle transport is impossible, and one would not expect the dispersion to enter the response functions. It implies that the factors of ω_{nm} in (8) can be absorbed into the wavefunctions. Below we show that it is indeed the case and can be done by taking the time dependence of states (or operators) into account. We start with the expression (10)

$$\sigma^{\mu\nu}_{\text{interband}}(\omega) = -\frac{ie^2}{\hbar}\sum_{n\neq m}\int_{\text{BZ}} f_n(1-f_m)\,\omega_{nm}\left(\frac{r^\mu_{nm}r^\nu_{mn}}{\omega_{nm}+\omega} - \frac{r^\nu_{nm}r^\mu_{mn}}{\omega_{nm}-\omega}\right). \tag{14}$$

We further decompose

$$\frac{\omega_{nm}}{\omega_{nm}+\omega} = 1 - \frac{\omega}{\omega_{nm}+\omega}\,, \qquad \frac{\omega_{nm}}{\omega_{nm}-\omega} = 1 + \frac{\omega}{\omega_{nm}-\omega}\,, \tag{15}$$

which yields

$$\sigma^{\mu\nu}_{\text{interband}}(\omega) = -\frac{e^2}{\hbar}\int_{\text{BZ}}\Omega_{\mu\nu} + \frac{ie^2\omega}{\hbar}\sum_{n\neq m}\int_{\text{BZ}} f_n(1-f_m)\left(\frac{r^\mu_{nm}r^\nu_{mn}}{\omega_{nm}+\omega} - \frac{r^\nu_{nm}r^\mu_{mn}}{\omega_{mn}+\omega}\right). \tag{16}$$

Reinstating the infinitesimal imaginary part of the frequency $\omega \to \omega_+$ to enforce convergence, and using the Schwinger representation

$$\frac{i}{\omega_{nm}+\omega_+} = \int_{-\infty}^{0} dt\, e^{-i(\omega_{nm}+\omega_+)t}\,, \tag{17}$$

one can show that the terms with propagators in (16) can be conveniently wrapped up as

$$\frac{e^2\omega}{\hbar}\sum_{n\neq m}\int_{\text{BZ}} f_n(1-f_m)\left(\int_{-\infty}^{0} dt\, e^{-i(\omega_{nm}+\omega_+)t} r^\mu_{nm}r^\nu_{mn} - \int_{-\infty}^{0} dt\, e^{-i(\omega_{mn}+\omega_+)t} r^\nu_{nm}r^\mu_{mn}\right) \tag{18}$$

$$= \frac{e^2\omega}{\hbar}\sum_{n\neq m}\int_{\text{BZ}} f_n(1-f_m)\left(\int_{-\infty}^{0} dt\, e^{-i\omega_+ t} r^\mu_{nm}r^\nu_{mn}(t) + \int_{0}^{-\infty} dt\, e^{-i\omega_+ t} r^\nu_{nm}(t)r^\mu_{mn}\right) \tag{19}$$

$$= \frac{e^2\omega}{\hbar}\int_{\text{C}} dt \int_{\text{BZ}} e^{-i\omega_+ t}\,\hat{T}Q^{\mu\nu}(t)\,, \tag{20}$$

where the integral is taken over the Keldysh contour C sketched in Figure 1, $\hat{T}$ denotes the (advanced) ordering of operators along C, and we introduced the time-dependent "quantum geometric tensor"

$$Q^{\mu\nu}(t) \equiv \text{Tr}\left[\hat{P}\hat{r}^\mu(0)(1-\hat{P})\hat{r}^\nu(t)\right], \tag{21}$$

where the operators appearing without the time label are taken at the initial time $t = 0$, and the trace runs over band indices. The interband contribution to the Kubo formula for conductivity, therefore, assumes the form

$$\sigma^{\mu\nu}_{\text{interband}}(\omega) = -\frac{e^2}{\hbar}\int_{\text{BZ}}\left(\Omega^{\mu\nu} - \omega\int_{\text{C}} dt\, e^{-i\omega_+ t}\,\hat{T}Q^{\mu\nu}(t)\right) = -i\frac{e^2}{\hbar}\int_{\text{BZ}}\int_{\text{C}} e^{-i\omega_+ t}\frac{d}{dt}\left(\hat{T}Q^{\mu\nu}(t)\right). \tag{22}$$

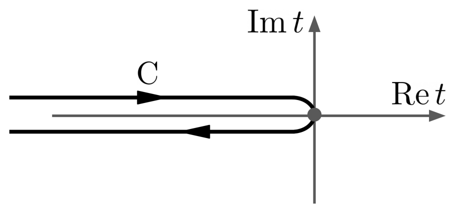

Supplementary Figure 1. Keldysh contour in complex time. The point at the axes crossing corresponds to $t = 0$.

As seen from the above, the time-dependent quantity $Q^{\mu\nu}(t)$ solely determines the interband part of the conductivity, as a function of the time-dependent quantum geometry. For example, in the case of Landau levels, we have $Q^{\mu\nu}(t) = Q^{\mu\nu}e^{i\omega_c t}$, and the momentum integral is taken over the reciprocal magnetic unit cell of the measure $2\pi/l_{\rm B}^2$. Plugging these expressions into Eq. (22), one obtains the ac conductivity tensor for Landau levels, Eq. (51). Hence, as proposed, the conductivity in insulators is entirely determined by the geometry of the Hilbert space.

Surprisingly, it turns out that not only the interband term allows for the geometric interpretation. In the next section, we take this reasoning one step further and show that the entire Kubo formula, including the intraband part, follows from the expectation value of the time-dependent position operator.

III. KUBO FORMULA FOR CONDUCTIVITY AS A DERIVATIVE OF THE POLARIZATION

In this section, we demonstrate that the schematic expression inspired by the modern theory of polarization [4]

$$\sigma^{\mu\nu} = \frac{\delta}{\delta E_\mu(t)} j^\nu(t) = \frac{\delta}{\delta E_\mu(t)} \frac{d}{dt} \langle P^\nu(t) \rangle \ , \tag{23}$$

is not merely a convenient symbolic way of introducing electrical conductivity. On the other hand, quite literally, (23) is the Kubo formula for conductivity. In order to show it, we write

$$\sigma^{\mu\nu} = \frac{\delta}{\delta E_\mu(t)} \frac{d}{dt} \left\langle \frac{-e\hat{r}^\nu(t)}{A} \right\rangle = -\frac{i\omega}{A} \frac{\delta}{\delta E_\mu(t)} \langle e\hat{r}^\nu(t) \rangle \ , \tag{24}$$

where the expectation value of the time-dependent position operator in the Heisenberg picture is obtained via the Schwinger-Keldysh formalism with the interaction Hamiltonian (note the second term required by hermiticity)

$$H_{\rm int} = J^\mu A_\mu = \frac{i}{\omega} J^\mu E_\mu e^{i\omega t} - \frac{i}{\omega} J^\mu E_\mu e^{-i\omega t} \, . \tag{25}$$

The vacuum expectation value of the position operator at time t is then

$$\begin{aligned} \frac{1}{A} \sum_n f_n \langle n | e\hat{r}^\nu(t) | n \rangle = \sum_n \int_{\rm BZ} f_n \langle n | \left(1 - \frac{1}{\hbar\omega} \int_{-\infty}^{t} {\rm d}t' \, e^{i\omega t'} \hat{J}_I^\mu(t') E_\mu \right) (e\hat{r}_I^\nu(t)) \times \\ \times \left(1 - \frac{1}{\hbar\omega} \int_{-\infty}^{t} {\rm d}t' \, e^{i\omega t'} \hat{J}_I^\sigma(t') E_\sigma \right) | n \rangle + {\rm c.c.} + O(E^2) \, , \end{aligned} \tag{26}$$

where the subscript I stands for the interaction picture with operators enjoying the "unperturbed" time-dependence $\hat{A}_{mn,I}(t) = e^{-i\omega_{mn} t} \hat{A}_I(0)$: in the following, to shorten the notation, we drop the subscript I. The expression c.c. stands for "complex conjugate" and contains the group of terms that oscillate with the frequency $e^{-i\omega t}$, which will be eliminated by the functional derivative $\delta/\delta E(t)$.

The $O(E^0)$ term in the expression (26) is related to the net Berry phase of the occupied bands

$$ie\hbar \sum_n \int_{\rm BZ} f_n \langle n | \partial^\mu n \rangle \ . \tag{27}$$

It corresponds to the ferroelectric polarization that material possesses in the absence of an external electric field. The remaining terms represent the dielectric response: the fluctuation of the electric dipole moment under the influence of the external field. We further omit the $O(E^2)$ terms that contain the information about the non-linear response: whether they reproduce the known non-linear contributions to conductivity is an interesting question that we leave for future work.

Keeping only the terms linear in E_ν, inserting another sum over energy eigenstates $\sum_n |m\rangle \langle m|$, and evaluating the time integrals, we write

$$\frac{1}{A} \sum_n f_n \langle n | e\hat{r}^\nu(t) | n \rangle = -\sum_{mn} \int_{\rm BZ} f_n \left(\frac{1}{\hbar\omega} \frac{e^{i(\omega+\omega_{nm})t}}{i(\omega_{nm}+\omega)} \langle n | \hat{J}^\mu(0) | m \rangle \, E_\mu \right) \langle m | e\hat{r}^\nu(0) | n \rangle \, e^{-i\omega_{nm} t} \tag{28}$$

$$-\sum_{mn} \int_{\rm BZ} f_n \langle n | e\hat{r}^\nu(0) | m \rangle \, e^{i\omega_{nm} t} \left(\frac{1}{\hbar\omega} \frac{e^{i(\omega-\omega_{nm})t}}{i(\omega-\omega_{nm})} \langle m | \hat{J}^\mu(0) | n \rangle \, E_\mu \right) + \ldots \tag{29}$$

The time dependence in this expression is canceled by the functional derivative, and we obtain

$$\frac{1}{A}\left(\frac{\delta}{\delta E_{\mu}(t)}\langle e\hat{r}^{\nu}(t)\rangle\right)=\frac{ie}{\hbar\omega}\sum_{mn}\int_{\mathrm{BZ}}f_n\left(\frac{J^{\mu}_{nm}r^{\nu}_{mn}}{\omega_{nm}+\omega}-\frac{r^{\nu}_{nm}J^{\mu}_{mn}}{\omega_{nm}-\omega}\right). \tag{30}$$

We then interchange the summation indices in the second bracket:

$$-\frac{i\omega}{A}\left(\frac{\delta}{\delta E_{\mu}}\langle e\hat{r}^{\nu}(t)\rangle\right)=\frac{e}{\hbar}\sum_{mn}\int_{\mathrm{BZ}}(f_n-f_m)\frac{J^{\mu}_{nm}r^{\nu}_{mn}}{\omega_{nm}+\omega}. \tag{31}$$

As soon as m and n are different, we are formally allowed to plug in $J^{\mu}_{nm}\to -ie\omega_{nm}r^{\mu}_{nm}$, so we arrive at the interband piece of the Kubo formula (8) :

$$\sigma^{\mu\nu}_{\mathrm{interband}}(\omega)=-\frac{ie^2}{\hbar}\sum_{n\neq m}\int_{\mathrm{BZ}}f_{nm}\omega_{nm}\frac{r^{\mu}_{nm}r^{\nu}_{mn}}{\omega_{nm}+\omega}. \tag{32}$$

In the case m and n are equal, we use the standard trick of splitting the initial and final states' momenta $|n\rangle\to|n\mathbf{k}'\rangle$, $|m\rangle\to|n\mathbf{k}\rangle$ and use the identity that follows from the distributional properties of the delta function

$$\langle n\mathbf{k}'|\hat{r}^{\mu}|n\mathbf{k}\rangle=-i\hbar\delta(\mathbf{k}-\mathbf{k}')\left(u_n^{\dagger}(\mathbf{k})\partial^{\mu}u_n(\mathbf{k})\right)+i\hbar\partial^{\mu}\delta(\mathbf{k}-\mathbf{k}'). \tag{33}$$

As we plug this expression into (31), the contribution from the first term containing a delta function vanishes after the momentum integration as $f_m\to f_n$. The the second term can be evaluated using integration by parts, which shifts the momentum derivative from the delta function to the Fermi distribution bringing about another factor of velocity. The resulting expression is nothing but the Drude intraband term (6)

$$\sigma^{\mu\nu}_{\mathrm{intraband}}(\omega)=\frac{i}{\omega}\sum_{n}\int_{\mathrm{BZ}}f'_nJ^{\mu}_{nn}J^{\nu}_{nn}. \tag{34}$$

Hence, Eq. (23) is indeed the Kubo formula for conductivity.

IV. RELATION BETWEEN TIME-DEPENDENT POLARIZATION AND CONDUCTIVITY IN LANDAU LEVELS

In this section, we illustrate how electrical conductivity can be obtained directly from the time-dependent electric dipole moment via the relation

$$\sigma^{\mu\nu}=-\frac{i\omega}{A}\frac{\delta}{\delta E_{\mu}(t)}\langle e\hat{r}^{\nu}(t)\rangle. \tag{35}$$

We perform this check for the Landau problem in oscillating in-plane electric field $E_x(t)=Ee^{i\omega t}$, where the matrix element $\langle\hat{r}^{\nu}(t)\rangle$ can be explicitly obtained.

It is convenient to choose the y-translationally-invariant gauge

$$A_x=\frac{i}{\omega}\mathcal{E}e^{i\omega t},\qquad A_y=Bx, \tag{36}$$

such that p_y is conserved. The time-dependent Schrödinger equation then assumes the form

$$\left[\frac{(\hat{p}_x+\frac{ie}{\omega}Ee^{i\omega t}+\mathrm{c.c.})^2}{2m_e}+\frac{(\hat{p}_y+eBx)^2}{2m_e}\right]\psi(x,y,t)=i\hbar\,\partial_t\psi(x,y,t). \tag{37}$$

One of the solutions is given by the gaussian ansatz

$$\psi_{p_y}(x,y,t)=\mathcal{N}(t)e^{ip_yy/\hbar}e^{-i\varphi(t)}\exp\left[-\frac{(x-x_c(t))^2}{2l_B^2}\right], \tag{38}$$

where $\varphi(t)$ is an energy phase inessential for the discussion, and

$$\begin{aligned} x_c(t) &= -\frac{p_y}{m_e\omega_c} + x_1(t) + ix_2(t)\,, \qquad & x_1(t) &= -\frac{2eE}{m_e}\frac{\cos(\omega t)}{\omega_c^2-\omega^2}\,, \\ x_2(t) &= \frac{2eE}{m_e}\frac{\omega_c}{\omega}\frac{\sin(\omega t)}{\omega_c^2-\omega^2}\,, & \mathcal{N}(t) &= \frac{1}{\sqrt{\sqrt{\pi}l_B}}e^{-x_2^2(t)/2l_B^2}\,. \end{aligned} \tag{39}$$

The wave function Eq. (38) should be understood as a time-dependent analog of the lowest Landau level. We then proceed to explicitly compute the matrix element of the position operator $\hat{x}$:

$$\begin{aligned} \langle\psi_{p_y}|\hat{x}|\psi_{p_y}\rangle &= \frac{1}{\sqrt{\pi}l_B}\int dx\, x\exp\left[-\frac{(x-x_1(t)+p_y/m_e\omega_c)^2}{l_B^2}\right] = \\ &= x_1(t) - p_y/m_e\omega_c = -\frac{eE}{m_e}\frac{1}{\omega_c^2-\omega^2}(e^{i\omega t}+e^{-i\omega t}) - p_y/m_e\omega_c\,. \end{aligned} \tag{40}$$

Taking the functional derivative (35), for the fully occupied Landau level ($A/N = \Phi_0/B = h/eB$) we obtain

$$\sigma^{xx} = i\frac{e^2}{h}\frac{\omega\omega_c}{\omega_c^2-\omega^2}\,, \tag{41}$$

in agreement with the result obtained from the Kubo formula in Appendix V. Remarkably, all we needed to extract this result is the expectation value of the position operator at the time t.

Unfortunately, our choice of gauge does not allow for the calculation of $\langle\hat{y}(t)\rangle$, since the wavefunction Eq. (38) does not decay in the y-direction. For completeness, however, we proceed by evaluating the current j^y, which is always well-defined:

$$\begin{aligned} j^y &= -\frac{e}{m_e}\int_{-\infty}^{\infty}\frac{dp_y}{2\pi\hbar}\psi_{p_y}^*(x,t)(p_y+eBx)\psi_{p_y}(x,t) \\ &= -\frac{e}{m_e}\int_{-\infty}^{\infty}\frac{dp_y}{2\pi\hbar}\psi_{p_y}^*(x,t)(m_e\omega_c x_1(t))\psi_{p_y}(x,t) = 2\frac{e^2}{h}\frac{\omega_c^2}{\omega_c^2-\omega^2}E\cos(\omega t)\,, \end{aligned} \tag{42}$$

from which we obtain

$$\sigma^{\mu\nu} = \frac{\delta j^\mu}{\delta E_\nu(t)} \;\longrightarrow\; \sigma^{xy}(\omega) = -\frac{e^2}{h}\frac{\omega_c^2}{\omega_c^2-\omega^2}\,. \tag{43}$$

In the stationary limit, this expression reduces to the well-known value for a fully occupied Landau level $\sigma_{xy} = -e^2/h$.

V. QUANTUM GEOMETRY AND CONDUCTIVITY IN LANDAU LEVELS

Consider the Hamiltonian for a two-dimensional electron gas in a uniform magnetic field

$$\hat{H}_{\rm LL} = \frac{\hat{\pi}^\mu\hat{\pi}_\mu}{2m_e}\,, \qquad \hat{\pi}^\mu = \hat{p}^\mu + e\hat{A}^\mu\,, \qquad B = \partial^x\hat{A}^y - \partial^y\hat{A}^x = \text{const}\,, \tag{44}$$

where $\hat{p}^\mu$ are the canonical momenta. The kinematical momenta $\hat{\pi}^\mu$, up to a normalization constant, commute canonically

$$[\hat{\pi}^\mu, \hat{\pi}^\nu] = -i\hbar eB\varepsilon^{\mu\nu}\,, \qquad \varepsilon^{\mu\nu} = \begin{pmatrix} 0 & 1 \\ -1 & 0 \end{pmatrix}\,, \tag{45}$$

which allows to define the interlevel ladder operators a, $a^\dagger$ with $a = (\pi^x - i\pi^y)/\sqrt{2\hbar eB}$. In order to compute quantum geometric quantities, it is convenient to have matrix elements of $\hat{r}^\mu$ to be well-defined. Hence, we adopt the radial gauge $A^\mu = -\varepsilon^{\mu\nu}x^\nu B/2$. It is then useful to define another set of momenta

$$\hat{\tilde{\pi}}^\mu = \hat{p}^\mu - e\hat{A}^\mu\,, \qquad [\hat{\tilde{\pi}}^\mu, \hat{\tilde{\pi}}^\nu] = i\hbar eB\varepsilon^{\mu\nu}\,, \tag{46}$$

that commute with any π^μ, and hence can be used to define ladder operators responsible for the degeneracy of the Landau levels. Using the definitions of π^μ and $\tilde{\pi}^\mu$, we express the coordinate operators in terms of the momenta

$$\hat{r}^\mu = \frac{l_B^2}{\hbar}\varepsilon^{\mu\nu}(\hat{\pi}^\nu - \hat{\tilde{\pi}}^\nu)\,. \tag{47}$$

The quantity we are interested in is the quantum geometric tensor (QGT) defined as

$$Q^{\mu\nu} = \mathrm{Tr}\left[\hat{P}\hat{r}^\mu\left(1-\hat{P}\right)\hat{r}^\nu\right] = \langle n-1|\hat{r}^\mu|n\rangle\,\langle n|\hat{r}^\nu|n-1\rangle\ , \tag{48}$$

where we accounted for the fact that only matrix elements taken between neighboring Landau levels are non-zero. Real and imaginary parts of the QGT are the quantum metric and Berry curvature

$$Q^{\mu\nu} = g^{\mu\nu} - \frac{i}{2}\Omega^{\mu\nu}\,. \tag{49}$$

Since the matrix element of $\hat{\tilde{\pi}}^\nu$ vanishes between states with the same momentum, we plug $\hat{\pi}^x = \sqrt{\hbar eB}(\hat{a}+\hat{a}^\dagger)/\sqrt{2}$, $\hat{\pi}^y = i\sqrt{\hbar eB}(\hat{a}-\hat{a}^\dagger)/\sqrt{2}$ into Eq. (48), and evaluate

$$\begin{aligned} Q^{\mu\mu} &= g^{\mu\mu} = \frac{l_B^2}{2}C\,, \\ Q^{xy} &= -\frac{i}{2}\Omega^{xy} = -i\frac{l_B^2}{2}C\,, \end{aligned} \tag{50}$$

where we identified the number of occupied Landau levels $n-1$ with a Chern number C. To determine conductivity, we use the expression for the conductivity tensor, Eq. (12) via the metric and Berry curvature, and utilize $\omega_{nm} = \delta_{n-1,m}\omega_c$, from which we immediately obtain the conductivity matrix

$$\sigma^{\mu\nu} = \frac{e^2}{h}C\frac{\omega_c^2}{\omega_c^2-\omega^2}\begin{pmatrix} i\omega/\omega_c & -1 \\ 1 & i\omega/\omega_c \end{pmatrix}\ , \tag{51}$$

where $\omega_c = eB/m_e$ is the cyclotron frequency. Inverting the above tensor, we find the resistivity matrix

$$\rho^{\mu\nu} = \frac{h}{e^2C}\begin{pmatrix} i\omega/\omega_c & 1 \\ -1 & i\omega/\omega_c \end{pmatrix}\ . \tag{52}$$

VI. CLASSICAL DERIVATION OF CAPACITANCE IN LANDAU LEVELS

It is well-known that many classical and quantum results obtained for the harmonic oscillator (and so, Landau levels) take the same form, and, as we will see, the result for the capacitance is no exception. In the following we briefly discuss this classical derivation of the longitudinal conductivity of the 2DEG in order to highlight the physical origin of this effect.

We consider classical free-electron gas in two dimensions subject to a perpendicular magnetic field as well as a harmonically oscillating electric force directed along the x-axis. The equation of motion of the individual electrons is

$$\ddot{x}(t) + \omega_c^2 x(t) = -\frac{e}{m_e}Ee^{i\omega t}\,. \tag{53}$$

The above equation is solved by the time-dependence

$$x(t) = -\frac{eEe^{i\omega t}}{m_e(\omega_c^2-\omega^2)}\,. \tag{54}$$

The x-polarization of the system is obtained as a net dipole moment per unit area

$$P(t) = \frac{N}{A}(-ex(t)) = \frac{N}{A}\frac{e^2}{m_e(\omega_c^2-\omega^2)}Ee^{i\omega t}\,. \tag{55}$$

The longitudinal conductivity is defined as

$$\sigma^{xx} = \frac{\delta}{\delta E(t)}\frac{d}{dt}P(t) = i\omega\frac{N}{A}\frac{e^2}{m_e(\omega_c^2-\omega^2)}\,. \tag{56}$$

To make a connection with the quantum result [Eq. (51)], we assume that the number of electrons N is just enough to occupy C Landau levels, i.e. $N\Phi_0/(AB) = C$, where $\Phi_0 = h/e$—flux quantum. Plugging N into (56), we obtain

$$\sigma^{xx} = i\frac{e^2 C}{h}\frac{\omega\omega_c}{\omega_c^2 - \omega^2}\,, \tag{57}$$

in full consistency with σ_{xx} found in Eq. (51).

VII. $2D$ GAPPED DIRAC CONE AND HALDANE MODEL

In this section, we discuss the capacitive conductivity of a two-dimensional gapped Dirac cone dispersion and its relation to the topology. We begin by considering the following Hamiltonian:

$$\hat{H} = k_x\hat{\sigma}_x + k_y\hat{\sigma}_y + M\hat{\sigma}_z\,. \tag{58}$$

The spectrum and the normalized eigenfunctions in this model are

$$E^{\pm} = \pm\sqrt{k^2+M^2}\,, \qquad u^{\pm}(\mathbf{k}) = \sqrt{\frac{E^+ \mp M}{2\omega^+}}\left(\frac{M \pm E^+}{k_x + ik_y}\,,\ \ 1\right)^T\,, \qquad k = \sqrt{k_x^2 + k_y^2}\,. \tag{59}$$

The diagonal components of the quantum metric can be found from $g^{\mu\mu} = |\langle u^+|\partial^\mu u^-\rangle|^2$, which gives

$$g^{xx} = \frac{k_y^2 + M^2}{4(k^2+M^2)^2}\,, \quad g^{yy} = \frac{k_x^2+M^2}{4(k^2+M^2)^2}\,, \quad g \equiv g^{xx} + g^{yy} = \frac{k^2+2M^2}{4(k^2+M^2)^2}\,. \tag{60}$$

Using the isotropy of the model (58), it is convenient to express the longitudinal conductivity as

$$\sigma^{xx} = \frac{\sigma^{xx}+\sigma^{yy}}{2} = \frac{ie^2}{\hbar}\int\frac{d^2k}{(2\pi)^2}\frac{2\omega\sqrt{k^2+M^2}}{4(k^2+M^2)-\omega^2}\frac{k^2+2M^2}{4(k^2+M^2)^2}\,, \tag{61}$$

where we utilized the formula (12). Before considering the insulating regime, $\omega < 2M$, it is instructive to analyze the $M \to 0$ case first. The integrand is necessarily singular in this limit, and one needs to re-introduce the scattering rate to stabilize the conductivity $\omega \to \omega + i\varepsilon$. The value of the integral is then well-defined, and σ^{xx} turns out to be purely real. Multiplying by four, which accounts for spin-valley degeneracy in graphene, we acquire the well-known result for the ac conductivity [5, 6]

$$\sigma^{xx} = \frac{\pi e^2}{2h}\,. \tag{62}$$

Remarkably, this contribution is both ω- and ε-independent.

In the opposite, insulating regime of vanishing frequency $\omega \to 0$ and a finite gap M, the system is characterized by a capacitive response $\sigma^{xx} = i\omega c_0$ with

$$c_0 = \frac{e^2}{12\pi|M|}\,. \tag{63}$$

This simple expression has wide applicability: it describes the universal value of capacitance arising from a single Dirac cone with the mass M. For topologically trivial materials like hexagonal boron nitride, c_0 multiplied by the number of Dirac cones provides a good estimate for the capacitance (cf. Fig. 2 in the main text).

On the other hand, unlike the Hall conductivity σ^{xy}, c_0 is insensitive to the sign of M, and so, is unable to track the band inversions and subsequent changes in topology: it also fails to capture the capacitance deep in the topological phase. In order to understand how the intrinsic capacitance is influenced by topology, one can introduce a parabolic correction to the Dirac cone Hamiltonian (58): $\hat{H}' = \hat{H} - \alpha k^2\hat{\sigma}_z$. This model is characterized by a unit Chern number in the parameter region $\alpha M > 0$. The quantum metric traced over the spatial indices in this model takes the form:

$$g = \frac{k^2 + 2M^2 + 2\alpha^2 k^4}{4(k^2+M^2-2k^2M\alpha+k^4\alpha^2)^2}\,. \tag{64}$$

The integral for conductivity can be performed analytically with the result for the capacitance

$$c_0' = \begin{cases} \frac{e^2}{12\pi}\frac{1}{|M||1-4M\alpha|}\,, & \alpha M < 0\,, \\ \frac{e^2}{12\pi|M|} + \frac{|\alpha|}{6\pi}\,, & \alpha M > 0\,. \end{cases} \tag{65}$$

Comparing this result with Eq. (63), we find good agreement in the trivial region where $\alpha M < 0$. However, in the topological region ($\alpha M < 0$) with Chern number ± 1, the extended Dirac cone result Eq. (65) does not decay to zero as $|M| \to \infty$, instead saturating at $|\alpha|/6\pi$, in strong contrast to Eq. (63). This reinforces the statement that the behavior of the intrinsic capacitance can serve as an indicator of the topology of the system.

Similar considerations apply to tight-binding models in a compact Brillouin zone. In order to illustrate this, we investigate a slight modification of the Haldane model

$$\hat{H}_{\mathrm{Haldane}} = -\frac{2}{3}(f(k_x,k_y)\hat{\sigma}_+ + f^*(k_x,k_y)\hat{\sigma}_-) + M\hat{\sigma}_z + M_{\mathrm{H}}\tilde{f}(k_x,k_y)\hat{\sigma}_z - \frac{3\sqrt{3}}{2}M_{\mathrm{H}}\hat{\sigma}_z\,, \tag{66}$$

where

$$\begin{aligned} f(k_x,k_y) &= \sum_i e^{-i\mathbf{a}_i\cdot\mathbf{k}}\,, \qquad && \mathbf{a}_1 = (0,-a),\ \mathbf{a}_{2,3} = (\pm\sqrt{3}a,a)/2\,, \\ \tilde{f}(k_x,k_y) &= \sum_i \sin\left(\mathbf{b}_i\cdot\mathbf{k}\right)\,, \qquad && \mathbf{b}_1 = (\sqrt{3}a,0)\,,\ \mathbf{b}_{2,3} = (-\sqrt{3}a,\pm 3a)/2\,, \end{aligned} \tag{67}$$

and we set for convenience $a = 1$. The last term in Eq. (66) is introduced only for ease of notation: It shifts the topological region in parameter space, such that for $M_{\mathrm{H}} > 0$, the parameter ranges $M < 0$ and $M > 3\sqrt{3}M_{\mathrm{H}}$ correspond to trivial phases. In the range $0 < M < 3\sqrt{3}M_{\mathrm{H}}$, the system acquires a finite Chern number $C = -1$. The resulting phase diagram, intrinsic capacitance and quantum metric are depicted in Fig. 2 of the main text.

VIII. DIELECTRIC CONSTANTS OF VARIOUS MATERIALS

The dielectric constant in crystalline materials receives contributions both from ionic and electronic degrees of freedom [7]. Here, We are focusing on the latter, eliminating the contribution due to lattice dynamics by assuming a high enough value of the driving frequency ω, while keeping it well below the gap Δ. In this regime, the "slow" lattice degrees of freedom remain inactive, whereas the electronic response remains entirely off-resonant, giving way to the quasi-static approximation assumed in Eq. (6) of the main text.

In the following, we describe how the dielectric constant can be connected with the intrinsic capacitance c. To this end, note that Eq. (6) in the main text, applied to the case of $3D$ materials, is closely related to the static dielectric susceptibility χ by a simple unit conversion factor of $\epsilon_0 \simeq 8.85\cdot 10^{-12}$ F/m. To see this, one expresses the longitudinal conductivity as

$$\sigma^{xx} = \frac{j^x}{E_x} = \frac{I^x}{A}\frac{d}{V_x} = \frac{d}{A}(i\omega C) = i\omega\frac{dC}{A} = i\omega\epsilon_0\chi\,, \tag{68}$$

where we assumed a rectangular slab-shaped insulator with thickness d and cross-section A, such that $C = \epsilon\epsilon_0\chi A/d$. On the other hand, $\sigma^{xx} = i\omega c$, which implies $\chi = c/\epsilon_0$. The value of the dielectric constant $\epsilon = 1+\chi$ is therefore given by the following linear response expression (see also [21]):

$$\epsilon = 1 + \frac{2e^2}{\hbar\epsilon_0}\sum_{m\neq n}\int_{\mathrm{BZ}} f_n(1-f_m)\frac{g^{xx}_{mn}}{\omega_{mn}}\,. \tag{69}$$

Note that by considering only the horizontal component of the metric g^{xx}, we restrict ourselves to the in-plane permittivity usually termed $\epsilon_{\|}$. Following [13], for ab initio calculations of ϵ using 2D tight-binding models, the integral over the vertical dimension of the Brillouin zone in (69) is replaced with the inverse monolayer thickness as

$$\int dk_z \to \frac{2\pi}{a_z}\,. \tag{70}$$

The experimental and theoretical values for the materials presented in Fig. 4 of the main text are tabulated in Supplementary Table 1. We point out that the electronic component of the dielectric constant in the literature is

Dielectric constants									
Material	Structure	Space group	Topology	ϵ_∞, exp.	ϵ_∞, th.	gap, eV	a_z, $\AA$	$2\pi\langle\bar{g}\rangle_z$, exp.	$2\pi\langle\bar{g}\rangle_z$, th.
Bi_2Se_3 [8, 9]	*rhombohedral*	R$\bar{3}$m	TI	29	34.7	0.3	9.84	2.87	3.45
Bi_2Te_3 [8, 9]	*rhombohedral*	R$\bar{3}$m	TI	85	101.7	0.13	10.5	3.98	4.77
Sb_2Te_3 [8]	*rhombohedral*	R$\bar{3}$m	TI	51	—	0.28	10.4	5.06	—
MoS_2 [10, 11]	*van der Waals*	P6_3/mmc	OAL[12]	16.4	18.0	1.82	3.13†	3.05	3.36
WS_2 [10, 11]	*van der Waals*	P6_3/mmc	OAL[12]	14.0	15.4	1.94	3.14†	2.75	3.05
$MoSe_2$ [10, 11]	*van der Waals*	P6_3/mmc	OAL[12]	17.6	19.1	1.51	3.35†	2.92	3.18
WSe_2 [10, 11]	*van der Waals*	P6_3/mmc	OAL[12]	15.8	16.2	1.59	3.36†	2.75	2.82
$MoTe_2$ [10, 11]	*van der Waals*	P6_3/mmc	OAL[12]	22.6	22.1	1.03	3.62†	2.80	2.73
hBN[13, 14]	*van der Waals*	P6_3/mmc	trivial	4.95	—	5.97	2.51†	2.06	—
CdTe[8]	*zincblende*	F$\bar{4}$3m	trivial	7.1	—	1.48	6.46	2.03	—
GaAs[8]	*zincblende*	F$\bar{4}$3m	trivial	10.9	—	1.42	5.65	2.76	—
InP[8]	*zincblende*	F$\bar{4}$3m	trivial	10.9	—	1.34	5.87	2.70	—
GaN[8]	*zincblende*	F$\bar{4}$3m	trivial	4.86	—	3.17	4.53	1.92	—
ZnSe[8]	*zincblende*	F$\bar{4}$3m	trivial	5.7	—	2.82	5.67	2.61	—
GaP[15]	*zincblende*	F$\bar{4}$3m	trivial	8.89	—	2.27	5.45	3.39	—
Si[15]	*diamond cubic*	Fd$\bar{3}$m	OAL	11.25	—	1.12	5.43	2.16	—
Ge[8]	*diamond cubic*	Fd$\bar{3}$m	OAL	16	—	0.81	5.66	2.39	—
C[15]	*diamond cubic*	Fd$\bar{3}$m	OAL	5.55	—	5.47	3.57	3.09	—
PbSe[8]	*rocksalt*	Fm$\bar{3}$m	trivial	22.9	—	0.28	6.12	1.30	—
PbTe[8, 16]	*rocksalt*	Fm$\bar{3}$m	trivial	34.8	—	0.31	6.46	2.35	—
SnTe[8, 17]	*rocksalt*	Fm$\bar{3}$m	TCI	55	—	0.36	6.33	4.27	—
MgO[8]	*rocksalt*	Fm$\bar{3}$m	trivial	2.94	—	7.9	4.22	2.25	—
LiF[18]	*rocksalt*	Fm$\bar{3}$m	trivial	1.92	—	14.2	4.03	1.83	—
NaCl[19]	*rocksalt*	Fm$\bar{3}$m	trivial	2.38	—	8.97	5.64	2.42	—
SiO_2 [19, 20]	*wurtzite*	P6_3mc	trivial	2.19	—	8.50	5.40	1.90	—
ZnO[19, 20]	*wurtzite*	P6_3mc	trivial	4.41	—	3.4	5.25	2.11	—
CaF_2 [19]	*fluorite*	Fm$\bar{3}$m	trivial	2.05	—	12.1	5.46	2.41	—

Supplementary Table 1. Electronic in-plane dielectric constants, values of the gap, and lattice constants a_z for selected semiconductors. The theoretical values of ϵ_∞ are obtained using the linear response expression (69). For transitional metal dichalcogenides, a 3-band tight-binding model [11] is used — the comparison of the $2D$ ab initio with the $3D$ experimental values is justified by the weak dependence of the dielectric constant on the number of layers [13]. The theoretical values for Bi_2Se_3 and Bi_2Te_3 are obtained using a tight-binding simulation with Slater-Koster parameters given in [9]. †For van der Waals materials, a_z refers to monolayer thickness, and the theoretical value of ϵ_∞ is computed for a monolayer.

commonly referred to as the *optical dielectric constant* or the *high-frequency dielectric constant*, and often denoted as ϵ_∞. For the sake of comparison with Fig. 4 of the main text, instead of using the rescaled susceptibility, we also plot the original product of the dielectric constant and gap ($\epsilon_\infty\Delta$) as a function of Δ in Supplementary Fig. 2. The difference is striking: while the materials belonging to different groups neatly align on $\langle\bar{g}\rangle_z$ axis, it is much harder to explain the trends in $\epsilon_\infty\Delta$ since the dielectric constant depends on the unit cell volume and symmetry, in addition to the influence of the quantum geometry which we explored in the main text.

Lastly, we analyze how well the value of $\langle\bar{g}\rangle_z$ obtained using the minimal gap Δ

$$\langle\bar{g}\rangle_z = \frac{\epsilon_0}{e^2} a_z \Delta(\epsilon - 1) \tag{71}$$

compares with the actual values of $\langle g\rangle_z$ obtained using the formula (14) in the main text using the tight-binding models [11, 22]. As one can infer from the first and third rows of Table 2, the values of $\langle g\rangle_z$ for topological insulators are much higher than the value of the lower bound, while for transitional metal dichalcogenides, the discrepancy is much milder. This difference can be explained by the fact that the topological insulators Bi_2Se_3 and Bi_2Te_3 are extremely dispersive, and the Brillouin zone integral in (69) receives most of the contributions from larger values of Δ. In order to obtain a better estimate of the quantum metric, some average value of the gap $\bar{\Delta}$ could be used. To this end, we write

$$\langle g\rangle_z \simeq \frac{\epsilon_0}{e^2} a_z \bar{\Delta}(\epsilon - 1)\,. \tag{72}$$

The most appropriate definition of $\bar{\Delta}$ is the gap at which the optical conductivity develops a maximum. This choice is motivated by the fact that the optical conductivity $\mathrm{Re}\,\sigma^{\mu\mu}$, a quantity proportional to the joint density of states, is related to the dielectric constant by a Kramers-Kronig relation

$$\epsilon^{\mu\mu} = 1 + \frac{2}{\pi\varepsilon_0}\int_0^\infty \frac{d\omega}{\omega^2}\mathrm{Re}\,\sigma^{\mu\mu}\,. \tag{73}$$

Because of the polynomial suppression of the high-frequency contribution in the integral above, good accuracy can be achieved by replacing $\hbar\omega$ under the integral with a gap $\bar{\Delta}$ corresponding to the first peak in the function $\mathrm{Re}\,\sigma^{\mu\mu}(\omega)$. Using this approximation with the expression for the real part of conductivity

$$\mathrm{Re}\,\sigma^{\mu\mu} = \frac{\pi e^2}{\hbar}\int_{\mathrm{BZ}}\sum_{n\neq m}\omega_{mn}\, g^{\mu\mu}_{mn}\delta(\omega-\omega_{mn})\,, \tag{74}$$

immediately reproduces the estimate (72). For example, such frequency $\bar{\Delta}$ corresponds to the gap at the M-point in transitional metal dichalcogenides, where a logarithmic van Hove singularity is located. The optical conductivity curves of both Bi_2Se_3 and Bi_2Te_3 have a single pronounced peak at $\bar{\Delta}\simeq 2\,\mathrm{eV}$ for the first and $\bar{\Delta}\simeq 1\,\mathrm{eV}$ for the second material [23, 24], and we use these values to estimate $\langle g\rangle_z$ with (72). As one can infer from the first and the fifth rows in Table 2, the values of the quantum metric obtained using (72) are in good agreement with the ab initio values (both are in bold). This shows that dielectric constants can be conveniently used to estimate the quantum metric once the average gap $\bar{\Delta}$ is identified from optical measurements or band structure arguments. Similar conclusion can be obtained for semiconductors, as shown in Table 3.

Material	MoS_2	WS_2	$MoSe_2$	WSe_2	$MoTe_2$	Bi_2Se_3	Bi_2Te_3
$2\pi\langle g\rangle_z$, theory	**5.00**	**5.08**	**4.89**	**4.93**	**4.78**	**18.3**	**29.9**
Δ (minimal gap)	1.82	1.94	1.51	1.59	1.03	0.3	0.13
$2\pi\langle\bar{g}\rangle_z$, experiment	3.05	2.75	2.92	2.75	2.80	2.87	3.98
$\bar{\Delta}$ (average gap)	2.72	3.44	2.29	2.89	1.74	2	1
$2\pi\langle g\rangle_z$, experiment	**4.55**	**4.88**	**4.42**	**4.99**	**4.72**	**19.1**	**30.6**

Supplementary Table 2. In the first row, the values of the quantum metric computed using the tight-binding models [9, 11]. The values of the minimal gap Δ and $\langle\bar{g}\rangle_z$ are copied from Table 1. The values of the energy gap at the M-point in the fourth row are found based on the tight-binding models for transitional metal dichalcogenides [11]. The estimates of the quantum metric obtained from experimental values of dielectric constants found in the fifth row are computed using (72).

Material	C	Si	Ge	GaAs	ZnSe
$2\pi\langle g\rangle_z$, theory[25]	**9.19**	**12.2**	**14**	**11.5**	**8.28**
Δ (minimal gap)	5.47	1.12	0.81	1.42	2.82
$2\pi\langle\bar{g}\rangle_z$, experiment	3.09	2.16	2.39	2.76	2.61
$\bar{\Delta}$ (average gap)[26]	13.6	4.8	4.3	5.2	7.1
$2\pi\langle g\rangle_z$, experiment	**7.67**	**9.28**	**12.7**	**10.1**	**6.57**

Supplementary Table 3. Same comparison as in Table 2, but for a selection of semiconductors. The theory values are based on the first-principles calculation of Ref. [25].

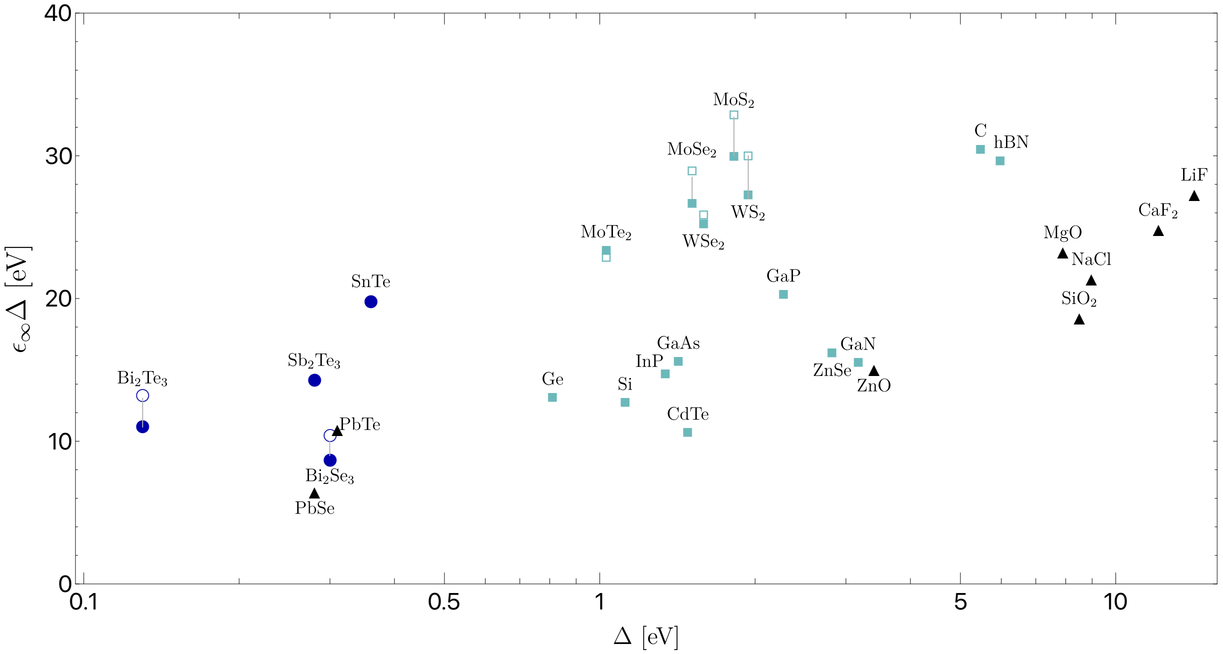

Supplementary Figure 2. Comparative figure demonstrating the conventional way of analyzing the dielectric constant as a function of the gap. Here we plot $\epsilon_\infty \Delta$, rather than $\langle \bar{g} \rangle_z = a_z \Delta(\epsilon_\infty - 1)$ (Fig. 4 of the main text). Note the absence of a trend: for instance, ionic trivial insulators denoted with black triangles span almost the entire range along the y-axis. In contrast, in Fig. 4 in the main text materials form tight groups.

[1] R. Kubo, Statistical-mechanical theory of irreversible processes. i. general theory and simple applications to magnetic and conduction problems, Journal of the Physical Society of Japan **12**, 570 (1957), https://doi.org/10.1143/JPSJ.12.570.

[2] B. Bradlyn, M. Goldstein, and N. Read, Kubo formulas for viscosity: Hall viscosity, Ward identities, and the relation with conductivity, Phys. Rev. B **86**, 245309 (2012), arXiv:1207.7021 [cond-mat.stat-mech].

[3] D. J. Thouless, M. Kohmoto, M. P. Nightingale, and M. den Nijs, Quantized hall conductance in a two-dimensional periodic potential, Phys. Rev. Lett. **49**, 405 (1982).

[4] R. Resta and D. Vanderbilt, Theory of polarization: A modern approach (2007) pp. 31–68.

[5] V. P. Gusynin, S. G. Sharapov, and J. P. Carbotte, Sum rules for the optical and hall conductivity in graphene, Phys. Rev. B **75**, 165407 (2007).

[6] S. H. Abedinpour, G. Vignale, A. Principi, M. Polini, W.-K. Tse, and A. H. MacDonald, Drude weight, plasmon dispersion, and ac conductivity in doped graphene sheets, Phys. Rev. B **84**, 045429 (2011).

[7] P. Bhattacharya, R. Fornari, and H. Kamimura, *Comprehensive semiconductor science and technology* (2011) pp. 1–647.

[8] O. Madelung, Semiconductors: Data handbook (2004).

[9] K. Kobayashi, Electron transmission through atomic steps of bi_2se_3 and bi_2te_3 surfaces, Phys. Rev. B **84**, 205424 (2011).

[10] B. Munkhbat, P. Wróbel, T. J. Antosiewicz, and T. Shegai, Optical constants of several multilayer transition metal dichalcogenides measured by spectroscopic ellipsometry in the 300-1700 nm range: high-index, anisotropy, and hyperbolicity, (2022), arXiv:2203.13793 [physics.optics].

[11] G.-B. Liu, W.-Y. Shan, Y. Yao, W. Yao, and D. Xiao, Three-band tight-binding model for monolayers of group-VIB transition metal dichalcogenides, Physical Review B **88**, 10.1103/physrevb.88.085433 (2013).

[12] J. Zeng, H. Liu, H. Jiang, Q.-F. Sun, and X. C. Xie, Multiorbital model reveals a second-order topological insulator in $1h$ transition metal dichalcogenides, Phys. Rev. B **104**, L161108 (2021).

[13] A. Laturia, M. L. Van de Put, and W. G. Vandenberghe, Dielectric properties of hexagonal boron nitride and transition metal dichalcogenides: from monolayer to bulk, npj 2D Materials and Applications **2**, 6 (2018).

[14] L. Wang, Y. Pu, A. K. Soh, Y. Shi, and S. Liu, Layers dependent dielectric properties of two dimensional hexagonal boron nitridenanosheets, AIP Advances **6**, 10.1063/1.4973566 (2016), 125126, https://pubs.aip.org/aip/adv/article-pdf/doi/10.1063/1.4973566/12989036/125126_1_online.pdf.

[15] D. Wing, G. Ohad, J. B. Haber, M. R. Filip, S. E. Gant, J. B. Neaton, and L. Kronik, Band gaps of crystalline solids from Wannier-localization-based optimal tuning of a screened range-separated hybrid functional, PNAS **118**, e2104556118 (2021), arXiv:2012.03278 [cond-mat.mtrl-sci].

[16] M. Baleva, E. Mateeva, and M. Momtchilova, The energy profile of polymorphous pbte films. i. direct energy gaps in pbte high-pressure phases and energies of the heterophase junctions, Journal of Physics: Condensed Matter **4**, 8997 (1992).

[17] K. Murase and S. Sugai, Raman scattering from soft to-phonon in iv–vi compound semiconductors, Solid State Communications **32**, 89 (1979).

[18] Datasheet from "pauling file multinaries edition – 2022" in springermaterials.

[19] *CRC Handbook of Chemistry and Physics* (2016).

[20] G. B. Grad, E. R. González, J. Torres-Díaz, and E. V. Bonzi, A DFT study of ZnO, Al2O3 and SiO2; combining X-ray spectroscopy, chemical bonding and Wannier functions, Journal of Physics and Chemistry of Solids **168**, 110788 (2022).

[21] G. Grosso and G. P. Parravicini, *Solid State Physics* (Academic Press, 2000).

[22] K. Kobayashi, T. Ohtsuki, K.-I. Imura, and I. F. Herbut, Density of States Scaling at the Semimetal to Metal Transition in Three Dimensional Topological Insulators, Phys. Rev. Lett. **112**, 016402 (2014), arXiv:1308.3953 [cond-mat.mes-hall].

[23] M. Eddrief, F. Vidal, and B. Gallas, Optical properties of Bi_2Se_3: from bulk to ultrathin films, Journal of Physics D Applied Physics **49**, 505304 (2016).

[24] D. Greenaway and G. Harbeke, Band structure of bismuth telluride, bismuth selenide and their respective alloys, Journal of Physics and Chemistry of Solids **26**, 1585 (1965).

[25] C. Sgiarovello, M. Peressi, and R. Resta, Electron localization in the insulating state: Application to crystalline semiconductors, Phys. Rev. B **64**, 115202 (2001).

[26] N. Ravindra, S. Auluck, and V. Srivastava, On the penn gap in semiconductors, physica status solidi (b) **93**, K155 (1979).
